# Prevalence and Predictors of Silent Myocardial Ischemia in Asymptomatic Type 2 Diabetes Mellitus: A Cross-Sectional Study From North India

**DOI:** 10.7759/cureus.108205

**Published:** 2026-05-03

**Authors:** Kashish Goyal, Deepak Sharma, Pankaj Bansal, Subhendu Mohanty, Manav Gandhi, Nivedita Sharma, Saket Kanodia, Siddharth Sahai

**Affiliations:** 1 Department of General Medicine, School of Medical Sciences and Research, Sharda University, Greater Noida, IND; 2 Department of Cardiology, School of Medical Sciences and Research, Sharda University, Greater Noida, IND

**Keywords:** cardiovascular risk factors, coronary artery disease, microalbuminuria, silent myocardial ischemia, treadmill stress test, type 2 diabetes mellitus

## Abstract

Background: Type 2 diabetes mellitus (T2DM) is associated with a substantially increased risk of cardiovascular complications, many of which remain silent until advanced stages. Silent myocardial ischemia (SMI), characterized by objective evidence of ischemia without anginal symptoms, is prevalent in diabetic populations due to autonomic neuropathy. Early identification of asymptomatic individuals harboring subclinical coronary artery disease (CAD) is crucial for timely intervention.

Objectives: This study aimed to determine the prevalence of coronary risk factors and SMI in asymptomatic patients with T2DM and to identify clinical and biochemical predictors associated with positive stress test findings.

Methods: This cross-sectional observational study enrolled 71 patients with T2DM (American Diabetes Association 2023 criteria) without known CAD at a tertiary care center in North India. All participants underwent treadmill stress testing (TMT) using the Bruce protocol. Demographic, lifestyle, clinical, and biochemical parameters were compared between TMT-positive (silent ischemia) and TMT-negative groups using chi-squared tests, Student's t-test, and Mann-Whitney U test as appropriate.

Results: SMI was detected in 18.31% (13/71) of participants. TMT-positive individuals demonstrated significantly higher prevalence of sedentary lifestyle (84.6% vs. 50%; p=0.011), diabetes duration >5 years (84.6% vs. 44.8%; p=0.004), hypertension (46.2% vs. 25.9%; p<0.001), smoking (76.9% vs. 44.8%; p=0.018), and family history of hypertension (69.2% vs. 31%; p=0.005). Biochemically, the TMT-positive group showed elevated serum urea (p=0.003), creatinine (p<0.001), triglycerides (p<0.001), low-density lipoprotein (LDL) (p=0.034), total cholesterol (p=0.002), and urinary albumin-to-creatinine ratio (UACR) (p<0.001) and lower high-density lipoprotein (HDL) (p<0.001).

Conclusion: A significant proportion of asymptomatic T2DM patients harbor SMI. Longer diabetes duration, sedentary lifestyle, hypertension, dyslipidemia, and microalbuminuria are key predictors. Routine cardiovascular screening using non-invasive modalities should be considered in high-risk diabetic individuals even without overt symptoms.

## Introduction

Diabetes mellitus, particularly type 2 diabetes mellitus (T2DM), represents one of the most pressing global health challenges of the 21st century, owing to its high and rising prevalence, chronic nature, and multifaceted complications. Defined by persistent hyperglycemia due to defects in insulin secretion, insulin action, or both, diabetes is not only a metabolic disorder but also a significant risk amplifier for cardiovascular morbidity and mortality [1].

The International Diabetes Federation estimated that approximately 537 million adults were living with diabetes in 2021, a figure projected to escalate to 783 million by 2045 [2]. This surge reflects a complex interplay of demographic, behavioral, and socioeconomic factors, including sedentary lifestyles, unhealthy diets, and increasing rates of obesity [3]. The most significant increases are expected in low- and middle-income countries, where rapid urbanization, sedentary lifestyles, and nutritional transitions have outpaced public health infrastructure [1]. In the Indian context, the burden is equally concerning. The National Noncommunicable Disease Monitoring Survey (NNMS) estimates the national prevalence of diabetes at 9.3%, reflecting both diagnosed and undiagnosed cases among adults aged 18-69 years [4]. The Indian Council of Medical Research-India Diabetes (ICMR-INDIAB) study further highlights considerable inter-state variability, with prevalence rates ranging from 4% to over 13% in different regions [5]. This epidemic imposes a substantial economic burden on healthcare systems and is recognized in the United Nations Sustainable Development Goals targeting the reduction of premature mortality from non-communicable diseases [6].

Individuals with T2DM face markedly elevated cardiovascular disease risk, which remains the leading cause of morbidity and mortality in this population [7]. The interplay of chronic hyperglycemia, insulin resistance, dyslipidemia, and endothelial dysfunction accelerates atherosclerosis, often beginning before clinical symptoms emerge [8]. Advanced glycation end products (AGEs), oxidative stress, and chronic inflammation damage the vascular endothelium and promote plaque formation [9]. Diabetic patients frequently exhibit clustering of traditional and non-traditional cardiovascular risk factors including hypertension, central obesity, elevated triglycerides, low high-density lipoprotein (HDL) cholesterol, and albuminuria [10]. 

A particularly concerning manifestation in T2DM is silent myocardial ischemia (SMI), where myocardial ischemia occurs without anginal symptoms or clinical manifestations [11]. This phenomenon is more prevalent in diabetics than in the general population, largely due to cardiovascular autonomic neuropathy impairing afferent nerve signaling and blunting ischemic pain perception [12]. Consequently, critical ischemic events may progress undetected until irreversible myocardial damage occurs. Studies have demonstrated that SMI is associated with adverse cardiovascular outcomes including sudden cardiac death and heart failure [13,14]. 

Despite substantial research linking diabetes to increased cardiovascular risk, the ability to accurately identify asymptomatic diabetic individuals harboring silent ischemia remains limited. While tools such as the Framingham Risk Score and other predictive models have been widely used to stratify cardiovascular risk, they often underperform in diabetic populations due to their failure to account for diabetes-specific pathophysiological mechanisms, such as autonomic neuropathy and microvascular disease [15]. Moreover, existing studies vary widely in methodology and screening modalities, resulting in inconsistent prevalence estimates. There is a clear need for population-specific data elucidating the burden of silent ischemia and delineating risk factors most strongly associated with its presence in T2DM, particularly in regions like India where diabetes prevalence is rising rapidly yet screening for silent coronary artery disease (CAD) remains limited.

Therefore, the present study aims to assess the prevalence of coronary risk factors and SMI in asymptomatic individuals with T2DM. By contrasting those with and without silent ischemia, this study seeks to identify key clinical and biochemical predictors, thereby contributing to more effective risk stratification and early intervention strategies tailored to this high-risk yet often overlooked subgroup [6]. 

## Materials and methods

Study design and setting

This was a prospective cross-sectional observational study conducted at Sharda Hospital, Greater Noida, India, over an 18-month period (April 2024 to November 2025). The study was approved by the Institutional Ethics Committee of the School of Medical Sciences and Research, Sharda University (approval number: SU/SMS&R/76-A/2024/71), and written informed consent was obtained from all participants.

Study population

Patients aged 35 years or older with clinically established T2DM based on American Diabetes Association (ADA) 2023 diagnostic criteria were enrolled consecutively [16]. 

Diagnostic Criteria for T2DM (≥35 Years of Age)

A diagnosis of diabetes can be made if any one of the following criteria is met: (1) glycated hemoglobin (HbA1c) ≥6.5% (the test should be performed in a laboratory using a method that is National Glycohemoglobin Standardization Program (NGSP)-certified and standardized to the Diabetes Control and Complications Trial (DCCT) assay), (2) fasting plasma glucose (FPG) ≥126 mg/dL (7 mmol/L) (fasting is defined as no caloric intake for at least eight hours), (3) two-hour plasma glucose ≥200 mg/dL (11.1 mmol/L) during an oral glucose tolerance test, or (4) random plasma glucose ≥200 mg/dL (11.1 mmol/L) (in a patient with classic symptoms of hyperglycemia or hyperglycemic crisis).

Inclusion Criteria

Included were (1) individuals with T2DM based on established parameters set forth by the ADA and (2) ≥35 years of age T2DM patients.

Exclusion Criteria

Excluded were (1) patients with signs and symptoms of CAD (patients with ongoing angina and baseline electrocardiogram (ECG) changes suggestive of CAD), displaying significant baseline ECG according to the Minnesota codes deviations such as elevated or depressed ST segments, abnormal T-wave patterns (inversion or flattening), presence of widened or deepened Q-waves, irregular heart rhythms, bundle branch blocks, or prolonged QT intervals, (2) patients with past treatment of CAD and congestive cardiac failure (CCF), and (3) patients with comorbidities such as renal disease, hepatic disease, ketosis, and stroke.

Sample Size Calculation

The sample size for the present study was calculated using Cochran's formula $\mathrm{n}_{0}=\mathrm{Z}^{2}\mathrm{pq}/\mathrm{e}^{2}$ for prevalence studies, where Z=1.96 at 95% confidence interval, p=4.8% (estimated prevalence of SMI among T2DM patients based on the ICMR-INDIAB study), q=1−p, and d=5% allowable error [5]. Using this formula, the calculated minimum sample size was 71 participants. Participants were recruited using a consecutive sampling technique until the required sample size was achieved.

Data collection

A comprehensive clinical history was obtained focusing on diabetes duration and management, hypertension, smoking habits, and alcohol consumption. The Modified Rose Angina Questionnaire, a validated epidemiological screening tool for ischemic cardiac symptoms, was utilized to screen for symptoms suggestive of angina or possible myocardial infarction [17]. SMI was defined as objective evidence of inducible myocardial ischemia on treadmill stress testing (TMT) in patients without prior clinical symptoms of angina as assessed using the Rose Angina Questionnaire. Clinical examination included brachial blood pressure measurement, anthropometry (height, weight, waist-to-hip ratio), and fundoscopy.

Laboratory investigations

Blood samples were collected after overnight fasting for fasting and postprandial blood glucose (glucose oxidase-peroxidase method), blood urea, serum creatinine, uric acid (colorimetric assays), lipid profile including total cholesterol, low-density lipoprotein (LDL), HDL, triglycerides, and very-low-density lipoprotein (VLDL) (enzymatic methods with Friedewald calculation for LDL/VLDL), and HbA1c by high-performance liquid chromatography. Urinary albumin-to-creatinine ratio (UACR) was calculated from early morning urine samples to detect microalbuminuria.

TMT

All participants underwent graded treadmill exercise testing following the standard Bruce protocol with continuous ECG, heart rate, and blood pressure monitoring. A positive test was defined as (1) horizontal or downsloping ST-segment depressions ≥1 mm or (2) test termination due to moderate-to-severe angina (substernal chest discomfort relieved with rest or nitroglycerin). Inconclusive tests were defined as negative ECG with submaximal exercise (failure to achieve average METs for age/sex or <85% maximum predicted heart rate) or early termination due to hypertensive response (systolic blood pressure >250 mmHg and/or diastolic blood pressure >115 mmHg) without meeting exercise thresholds. Two-dimensional echocardiography was performed to exclude non-coronary causes of ST changes.

Based on TMT results, participants were categorized into the non-CAD group (negative stress test) and the CAD group (positive test indicating SMI).

Statistical analysis

Data were analyzed using IBM SPSS Statistics for Windows, Version 26.0 (IBM Corp., Armonk, New York, United States). Continuous variables were expressed as mean±standard deviation. Categorical variables were compared using the chi-squared test, with odds ratios and relative risk calculated. For normally distributed continuous variables, Student's t-test was used; the Mann-Whitney U test was applied for non-normally distributed variables. One-way ANOVA was employed for multiple group comparisons. A p-value of <0.05 was considered statistically significant.

**Figure 1:**
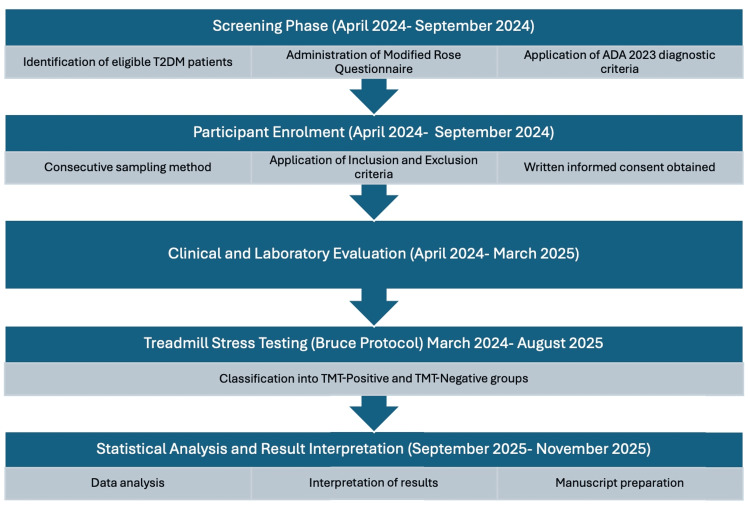
Illustrative study timeline of the prospective observational study Illustrative study timeline showing participant eligibility assessment, enrollment using consecutive sampling, clinical and laboratory evaluation, treadmill stress testing using the Bruce protocol, and final statistical analysis phases conducted during the 18-month prospective study period (April 2024 to November 2025). Figure created using Microsoft PowerPoint (Microsoft Corporation, Redmond, Washington, United States) T2DM: type 2 diabetes mellitus; ADA: American Diabetes Association; TMT: treadmill stress testing

**Figure 2:**
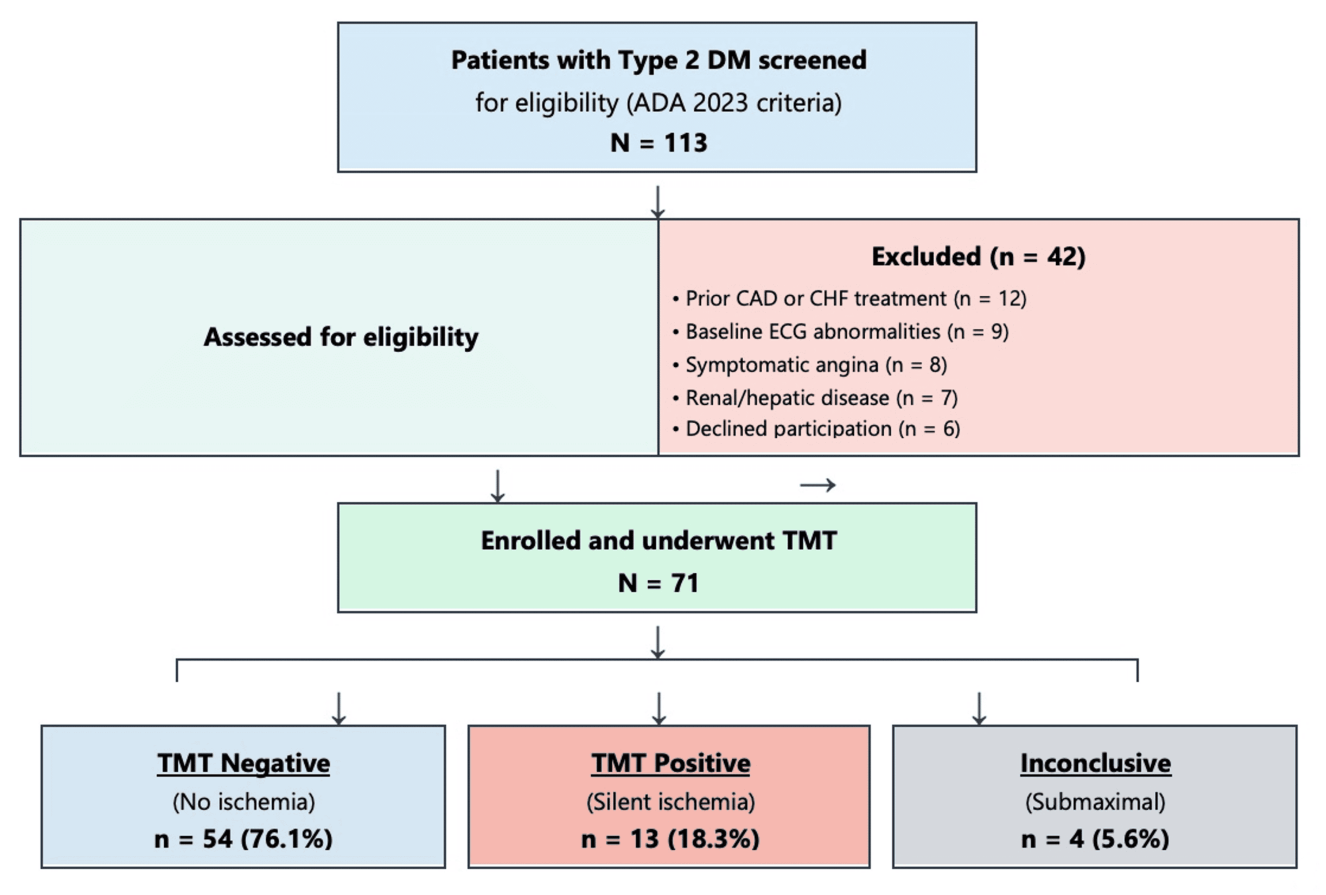
Participant recruitment flowchart and TMT outcomes Positive TMT is defined as horizontal or downsloping ST-segment depression ≥1 mm or test termination due to moderate-to-severe angina. Figure generated using Microsoft Word (Microsoft Corporation, Redmond, Washington, United States) DM: diabetes mellitus; ADA: American Diabetes Association; CAD: coronary artery disease; CHF: congestive heart failure; ECG: electrocardiogram; TMT: treadmill stress testing

Figure 1 depicts the timeline of the prospective observational study, while Figure 2 shows the participant recruitment flowchart and TMT outcomes.

## Results

Participant characteristics

A total of 71 participants with T2DM meeting the eligibility criteria were enrolled. The mean age was 53.21±9.73 years (median 53; IQR 16 years). The majority were male (80.3%; n=57), with 52.1% residing in rural areas. Regarding occupation, 32.4% were employed in government organizations, 26.8% were employed in the private sector, 15.5% were unemployed, 14.1% were self-employed, and 11.3% were homemakers. A sedentary occupational history was reported by 56.3% of participants.

Half of the participants (52.1%) had diabetes duration <5 years, while 47.9% had diabetes for >5 years. Treatment comprised oral hypoglycemic agents in 70.4% and insulin therapy in 29.6%. Hypertension was present in 29.6% (n=21). Family history was positive for diabetes in 49.3%, hypertension in 38.1%, and obesity in 57.7%. Alcohol consumption was reported by 60.5% and smoking by 50.7%. Dietary habits included vegetarian (49.3%), non-vegetarian (26.8%), and mixed diet (23.9%). Baseline characteristics are presented in Table 1.

**Table 1:** Baseline demographic, clinical, and lifestyle characteristics of the study participants (N=71) Categorical variables are presented as n (%) and continuous variables as mean±standard deviation (SD). Comparisons between groups were performed using the chi-squared test for categorical variables and Student's t-test for continuous variables.

Characteristic	n (%)	Mean±SD
Age (years)	-	53.21±9.73
Male sex	57 (80.3)	-
Rural residence	37 (52.1)	-
Sedentary occupation	40 (56.3)	-
Diabetes duration >5 years	34 (47.9)	-
Insulin therapy	21 (29.6)	-
Hypertension	21 (29.6)	-
Family history of diabetes	35 (49.3)	-
Family history of hypertension	27 (38.1)	-
Family history of obesity	41 (57.7)	-
Smoking	36 (50.7)	-
Alcohol consumption	43 (60.5)	-

Prevalence of SMI

**Figure 3:**
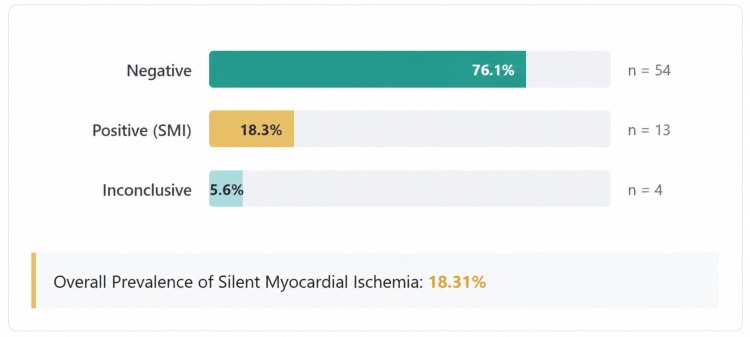
Distribution of TMT results among the study participants Distribution of TMT results among the study participants showing proportions of TMT-negative individuals, TMT-positive cases suggestive of SMI, and inconclusive results. The overall prevalence of SMI in the study population was 18.31%. Figure generated using Python (Version 3.1, Python Software Foundation, Wilmington, Delaware, United States) with the Matplotlib library (Version 3.1) and exported at 300 DPI resolution in PNG format TMT: treadmill stress testing; SMI: silent myocardial ischemia

TMT revealed that 54 participants (76.1%) had negative results, 13 (18.3%) had positive results indicating SMI, and four (5.6%) had inconclusive results. Thus, the prevalence of SMI among asymptomatic T2DM patients was 18.31% (Figure 3).

Comparison of risk factors by TMT status

Table 2 presents the comparison of demographic, lifestyle, and clinical factors between TMT-positive and TMT-negative groups. Age did not differ significantly between groups (55.85±11.13 vs. 52.62±9.4 years; p=0.283). Sex distribution was similar (p=0.736).

**Table 2:** Comparison of demographic and lifestyle factors between TMT-positive and TMT-negative groups Categorical variables are presented as n (%) and continuous variables as mean±standard deviation (SD). The chi-squared test was used for categorical variables and Student's t-test for continuous variables. *: statistically significant values defined as p<0.05 DM: diabetes mellitus; TMT: treadmill stress testing; HTN: hypertension

Variable	TMT-positive group (n=13)	TMT-negative group (n=58)	P-value
Age (years), mean±SD	55.85±11.13	52.62±9.4	0.283
Male sex, n (%)	10 (76.9)	47 (81)	0.736
Sedentary occupation, n (%)	11 (84.6)	29 (50)	0.011*
DM duration >5 years, n (%)	11 (84.6)	26 (44.8)	0.004*
Hypertension, n (%)	6 (46.2)	15 (25.9)	<0.001*
Smoking, n (%)	10 (76.9)	26 (44.8)	0.018*
Alcohol consumption, n (%)	11 (84.6)	32 (55.2)	0.024*
Family history of diabetes, n (%)	8 (61.5)	27 (46.6)	0.069
Family history of HTN, n (%)	9 (69.2)	18 (31)	0.005*
Family history of obesity, n (%)	11 (84.6)	30 (51.7)	0.015*

Sedentary occupational history was significantly more prevalent in the TMT-positive group (84.6% vs. 50%; p=0.011). Diabetes duration >5 years was strongly associated with positive TMT (84.6% vs. 44.8%; p=0.004). History of hypertension showed a significant association (46.2% vs. 25.9%; p<0.001).

Among lifestyle factors, smoking was significantly more common in TMT-positive individuals (76.9% vs. 44.8%; p=0.018), as was alcohol consumption (84.6% vs. 55.2%; p=0.024). Family history of hypertension demonstrated a strong association with positive TMT (69.2% vs. 31%; p=0.005), while family history of diabetes showed borderline significance (61.5% vs. 46.6%; p=0.069). Family history of obesity was also significantly more prevalent in the TMT-positive group (84.6% vs. 51.7%; p=0.015). Dietary pattern showed no significant association (p=0.939).

Biochemical parameters

Table 3 presents the comparison of laboratory parameters between groups. TMT-positive patients demonstrated significantly elevated renal markers: serum urea (49.46±3.56 vs. 31.11±3.64 mg/dL; p=0.003) and creatinine (1.2±0.19 vs. 0.9±0.09 mg/dL; p<0.001). Serum uric acid was also significantly higher (7.04±0.4 vs. 5.89±0.41 mg/dL; p=0.012).

**Table 3:** Comparison of biochemical parameters between TMT-positive and TMT-negative groups Values are expressed as mean±standard deviation (SD). Student's t-test was used for normally distributed variables and the Mann-Whitney U test for non-normally distributed variables. *: statistically significant values defined as p<0.05 TMT: treadmill stress testing; LDL: low-density lipoprotein; HDL: high-density lipoprotein; HbA1c: glycated hemoglobin; UACR: urinary albumin-to-creatinine ratio; TSH: thyroid-stimulating hormone; TLC: total leukocyte count

Parameter	TMT-positive group (n=13)	TMT-negative group (n=58)	P-value
Serum urea (mg/dL)	49.46±3.56	31.11±3.64	0.003*
Serum creatinine (mg/dL)	1.2±0.19	0.9±0.09	<0.001*
Serum uric acid (mg/dL)	7.04±0.4	5.89±0.41	0.012*
Triglycerides (mg/dL)	219.65±28.25	158.53±21.26	<0.001*
Total cholesterol (mg/dL)	245.61±19.96	196.51±11.41	0.002*
LDL cholesterol (mg/dL)	150.81±19.59	108.89±12.5	0.034*
HDL cholesterol (mg/dL)	35.41±3.31	49.18±6.37	<0.001*
HbA1c (%)	8.85±0.47	7.13±0.36	0.084
UACR (mg/g)	274.03±63.5	77.61±32.01	<0.001*
TSH (µIU/mL)	4.16±1.06	3.18±0.87	0.006*
Hemoglobin (g/dL)	11.06±1.2	12.84±0.9	<0.001*
TLC (×10³/µL)	9.24±1.5	7.49±1.2	<0.001*
Platelet count (×10³/µL)	335±45	275±38	<0.001*

The lipid profile reflected more adverse cardiovascular risk in the TMT-positive group: triglycerides (219.65±28.25 vs. 158.53±21.26 mg/dL; p<0.001), total cholesterol (245.61±19.96 vs. 196.51±11.41 mg/dL; p=0.002), and LDL cholesterol (150.81±19.59 vs. 108.89±12.5 mg/dL; p=0.034). HDL cholesterol was significantly lower (35.41±3.31 vs. 49.18±6.37 mg/dL; p<0.001).

**Figure 4:**
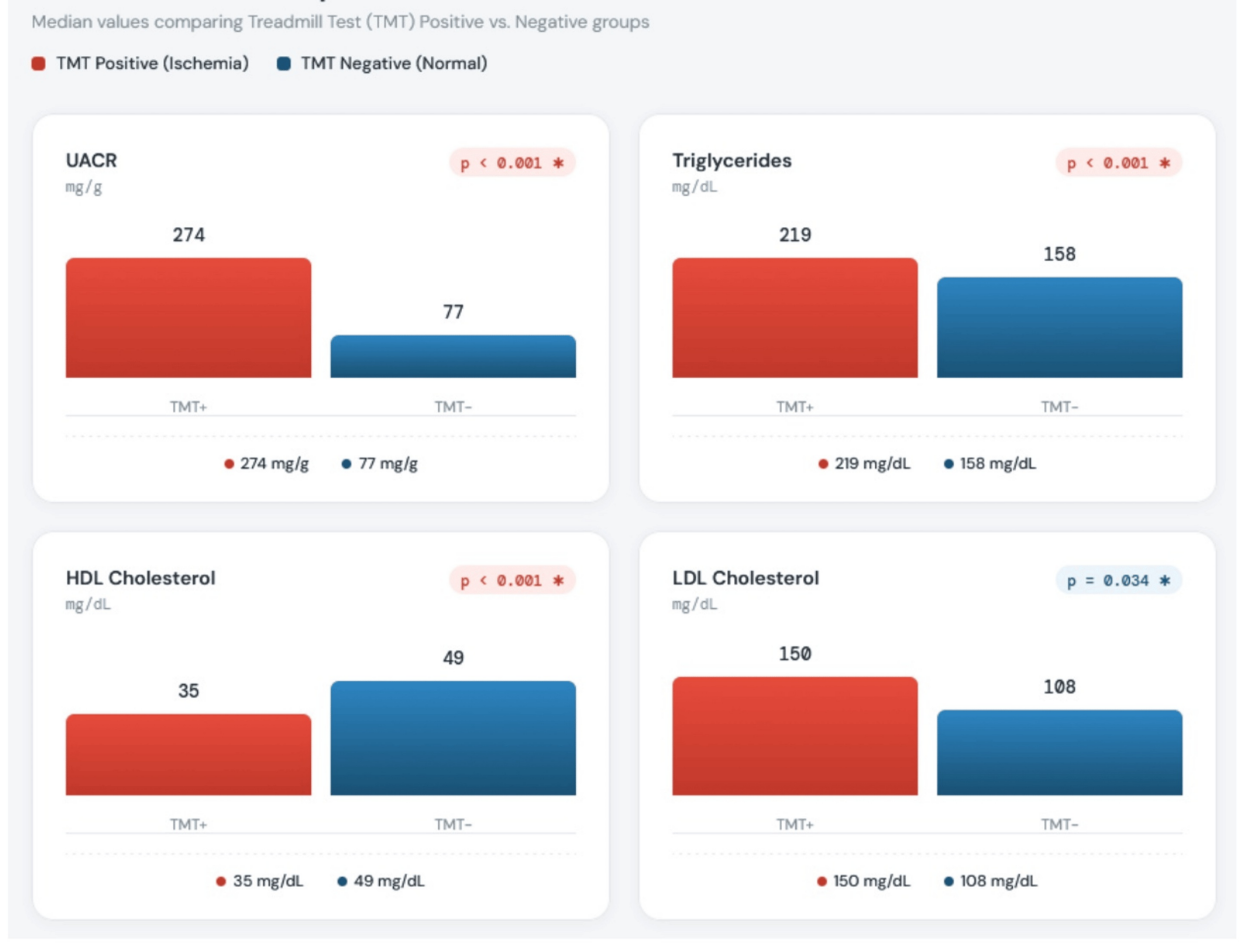
Comparison of selected biochemical parameters between TMT-positive and TMT-negative groups. Box plots show median, interquartile range, and outliers Figure generated using Python (Version 3.1, Python Software Foundation, Wilmington, Delaware, United States) with the Matplotlib library (Version 3.1) and exported at 300 DPI resolution in PNG format TMT: treadmill stress testing; UACR: urinary albumin-to-creatinine ratio; HDL: high-density lipoprotein; LDL: low-density lipoprotein

UACR, a marker of early nephropathy and vascular injury, was markedly elevated in the TMT-positive group (274.03±63.5 vs. 77.61±32.01 mg/g; p<0.001). HbA1c was higher in the TMT-positive group (8.85±0.47% vs. 7.13±0.36%) though this did not reach statistical significance (p=0.084). Thyroid-stimulating hormone (TSH) was significantly elevated in the TMT-positive group (4.16±1.06 vs. 3.18±0.87 µIU/mL; p=0.006) (Figure 4).

## Discussion

**Figure 5:**
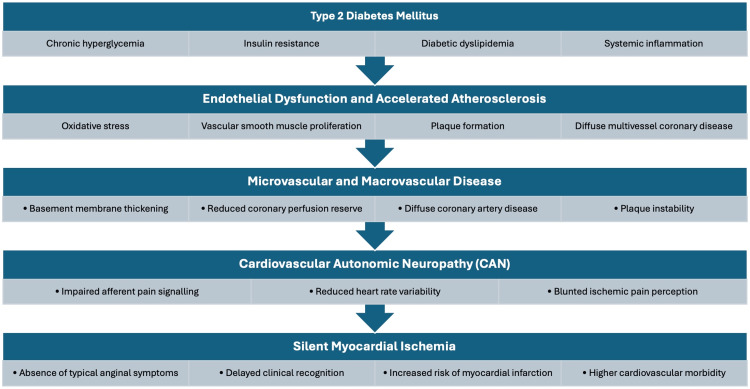
Pathophysiological mechanisms underlying silent myocardial infarction in type 2 diabetes mellitus Schematic representation of the pathophysiological mechanisms underlying silent myocardial ischemia in type 2 diabetes mellitus. Chronic hyperglycemia and insulin resistance lead to endothelial dysfunction, accelerated atherosclerosis, and cardiovascular autonomic neuropathy, resulting in impaired ischemic pain perception and delayed recognition of myocardial ischemia. Figure created using Microsoft PowerPoint (Microsoft Corporation, Redmond, Washington, United States)

The present study was undertaken with the primary objective of assessing the prevalence and distribution of coronary risk factors among patients with T2DM in the absence of overt coronary heart disease. Cardiovascular disease remains the leading cause of morbidity and mortality in patients with T2DM, and a significant proportion of these individuals may develop SMI or asymptomatic myocardial ischemia, which often goes unrecognized until advanced disease or major adverse events occur (Figure 5).

The study revealed that 18.31% of asymptomatic patients with T2DM demonstrated electrocardiographic evidence of SMI during TMT. This finding underscores the substantial hidden burden of coronary disease in diabetic individuals who lack overt symptoms, reinforcing the importance of proactive cardiovascular screening in this high-risk population.

The prevalence observed in our study aligns closely with previous investigations. Rokicka et al. reported SMI in 32.4% of patients with T2DM compared to 17.9% in type 1 diabetes, with contributing factors including advancing age, carotid atherosclerosis, and reduced left ventricular ejection fraction [18]. Giovacchini et al. found a 32% prevalence using myocardial perfusion imaging in asymptomatic T2DM patients [19]. Prasad et al. estimated approximately 25% of asymptomatic diabetic patients exhibit silent ischemia, identifying age, carotid intima-media thickness, hypercholesterolemia, and hypertriglyceridemia as significant predictors [14]. The JPAD trial by Soejima et al. demonstrated that SMI accounted for 25% of all myocardial infarctions in diabetic patients over a decade of follow-up [20]. The slightly lower prevalence in our study may reflect differences in diagnostic modality (TMT vs. myocardial perfusion imaging), population characteristics, or selection criteria.

Lifestyle and behavioral factors

The present study demonstrated several significant associations between lifestyle factors and TMT positivity, which serves as a proxy for silent CAD among patients with T2DM. These associations underscore the intricate interplay between behavioral habits, family history, and cardiovascular risk, even in asymptomatic diabetic individuals.

Sedentary occupational history emerged as a significant predictor of TMT positivity (84.6% vs. 50%; p=0.011). This finding is consistent with Swarnkar et al., who emphasized that sedentary occupation compounded by poor dietary choices significantly increased subclinical atherosclerosis risk in T2DM [21]. Davalagi et al. similarly found that physical inactivity was significantly higher among T2DM patients with elevated cardiovascular risk scores in an urban slum population in Karnataka [22]. Physical inactivity contributes to insulin resistance, obesity, dyslipidemia, and chronic inflammation, all amplifying cardiovascular risk.

Smoking (76.9% vs. 44.8%; p=0.018) and alcohol consumption (84.6% vs. 55.2%; p=0.024) were significantly more prevalent in TMT-positive individuals. Ulambayar et al. found smoking independently associated with increased 10-year cardiovascular risk among adults with diabetes in a Hungarian population-based study [23]. These modifiable risk behaviors highlight the critical importance of lifestyle counseling as a primary preventive strategy in diabetic care.

Clinical predictors

Diabetes duration >5 years was strongly associated with positive TMT findings (84.6% vs. 44.8%; p=0.004). This aligns with de Jong et al., who demonstrated that each additional five years of diabetes duration significantly increased major cardiovascular event risk [24]. Chronic hyperglycemia over extended periods induces endothelial dysfunction, promotes oxidative stress, and accelerates atherosclerosis. 

Hypertension showed a significant association with silent ischemia (46.2% vs. 25.9%; p<0.001). This is supported by Rawshani et al., who identified hypertension as a key modifiable risk factor contributing to increased mortality and cardiovascular morbidity in both type 1 and type 2 diabetes in a nationwide Swedish registry [25]. Hypertension amplifies cardiovascular risk through arterial stiffness, left ventricular hypertrophy, and increased myocardial oxygen demand.

Family history of hypertension demonstrated a strong association (69.2% vs. 31%; p=0.005), suggesting potential genetic and familial clustering of cardiometabolic risk that may predispose to early silent CAD. Stojanović et al. reported that patients with a family history of hypertension demonstrated greater prevalence of cardiovascular complications and poorer health-related quality of life [26].

Biochemical predictors

UACR emerged as one of the most potent predictors, with TMT-positive patients showing more than threefold higher levels (274.03 vs. 77.61 mg/g; p<0.001). This aligns with Giovacchini et al., who found microalbuminuria to be the only significant predictor of silent ischemia with 71.4% overall accuracy [19]. Microalbuminuria reflects generalized endothelial dysfunction and systemic vascular pathology, serving as a proxy for widespread vascular damage including the coronary vasculature [13].

The atherogenic dyslipidemic profile observed, namely, elevated LDL, triglycerides, and total cholesterol with reduced HDL, is characteristic of diabetic dyslipidemia and strongly implicated in coronary plaque formation. Bertoluci and Rocha emphasized that atherogenic dyslipidemia, particularly elevated triglycerides and low HDL, is more predictive of CAD in diabetics than LDL alone due to increased small dense LDL particles [8].

Elevated TSH in the TMT-positive group (4.16 vs. 3.18 µIU/mL; p=0.006) suggests subclinical hypothyroidism as a potential contributor. Subclinical hypothyroidism has been increasingly linked to atherosclerotic changes through lipid metabolism dysregulation and endothelial dysfunction [27].

Clinical implications

The identification of 18.31% silent ischemia prevalence in seemingly low-risk asymptomatic diabetics emphasizes the urgent need for proactive screening strategies. Non-invasive screening using TMT can significantly alter diagnostic and therapeutic trajectories when applied to high-risk individuals. The identification of multiple predictors, including longer diabetes duration, sedentary lifestyle, hypertension, dyslipidemia, and microalbuminuria, provides a pragmatic framework for selecting patients warranting early diagnostic testing, tailored risk assessment, and timely interventions.

Behavioral interventions emphasizing physical activity, smoking cessation, and alcohol moderation are indispensable not only for glycemic control but also for vascular protection. Routine UACR screening should be integrated into annual diabetes assessments, with elevated values prompting the escalation of cardiovascular surveillance and therapeutic intensification including renin-angiotensin-aldosterone system (RAAS) blockers and sodium-glucose cotransporter 2 (SGLT2) inhibitors.

Moreover, integrating validated risk models such as the Framingham or UKPDS Risk Engines, tailored to local population characteristics, can assist clinicians in estimating individual patient risk and setting therapeutic targets. These tools, in combination with laboratory surveillance and lifestyle intervention, can substantially reduce the burden of undiagnosed and untreated silent ischemia [28]. 

Strengths and limitations

Strengths of this study include the targeted evaluation of silent ischemia in a clinically relevant yet under-investigated population, comprehensive assessment of multiple variables, use of standardized TMT protocol, and focus on an Indian population enhancing regional relevance.

Limitations include the relatively small sample size restricting generalizability and statistical power. The relatively small number of positive stress test cases limited the ability to perform adjusted multivariable analysis. The cross-sectional design prevents the establishment of causal relationships. The absence of a non-diabetic comparator group limits the assessment of the incremental contribution of diabetes mellitus to inducible myocardial ischemia risk. Reliance on TMT alone may lead to underdiagnosis compared to myocardial perfusion imaging. Exercise treadmill testing is a screening modality and does not provide definitive anatomical confirmation of CAD. Some relevant markers, such as high-sensitivity C-reactive protein (CRP) and carotid intima-media thickness, were not assessed. As a single-center hospital-based study, selection bias may influence participant characteristics. 

Future directions of the study

Future multicentric prospective studies with larger sample sizes are required to validate the predictors of SMI identified in the present study across diverse populations. Incorporation of advanced imaging modalities such as myocardial perfusion imaging, coronary CT angiography, and stress echocardiography is warranted to further validate predictors of SMI. Future research may also explore the role of emerging biomarkers and structured cardiometabolic risk scores in enhancing risk stratification and guiding personalized screening strategies in patients with T2DM. 

## Conclusions

This study demonstrates a significant prevalence of SMI (18.31%) among patients with T2DM who exhibit no overt symptoms of coronary heart disease. Several clinical and biochemical variables, including longer diabetes duration, sedentary lifestyle, hypertension, dyslipidemia, and microalbuminuria, showed significant associations with treadmill-detected SMI in this study. Laboratory biomarkers, including deranged renal function, altered lipid profile, and elevated inflammatory markers, further delineate the higher risk burden in TMT-positive individuals. These findings support the role of early cardiovascular risk stratification using non-invasive tools such as TMT in selected high-risk diabetic patients and highlight the importance of integrating structured cardiovascular risk stratification into routine diabetes care to improve long-term outcomes.
